# Danish Evaluation of Your Heart Forecast (DANY): study protocol for a cluster randomised controlled trial on an interactive risk-communication tool aimed at improving adherence of patients with high blood pressure

**DOI:** 10.1186/s13063-019-3886-2

**Published:** 2020-01-03

**Authors:** Anders Elkær Jensen, Jens Søndergaard, Niels Kristian Kjær, Rod Jackson, Jesper Bo Nielsen

**Affiliations:** 1 Research Unit of General Practice, Department of Public Health, University of Southern Denmark, J.B. Winsløws Vej 9A, 5000 Odense, Denmark; 2 Faculty of Medical and Health Sciences, University of Auckland, Auckland, New Zealand

**Keywords:** CVD risk, Cardiovascular risk, Heart age, Interactive, Dynamic, Risk communication, General practice, Primary care, Your Heart Forecast, Protocol

## Abstract

**Background:**

To improve communication of risk messages, they must be communicated in a way that is understandable and relevant to the patient. Communicating risk of cardiovascular disease is a complex and individualised task, since the risk itself is a combination of multiple personal risk factors. Raised blood pressure is but one of these risk factors. In Denmark, only one-third of hypertensive patients are adequately treated, with regards to national clinical guidelines. One reason for this problem is low treatment adherence; tools with documented effects for increasing adherence of patients are limited.

Our objective is to evaluate the effect of a personalised, interactive and dynamic risk-assessment and risk-communication tool: ‘Your Heart Forecast’ (YHF) on blood pressure control, primary non-compliance, health literacy and patient empowerment.

**Methods:**

Cluster-randomised controlled trial in general practice. Effect measures are adherence, blood pressure, lipid levels and empowerment at inclusion and after 6 and 12 months. To identify other benefits or possible adverse effects of the intervention, qualitative interviews will be conducted with a subgroup of patients.

**Discussion:**

The investigators will explore effects of Your Heart Forecast on patients’ health literacy, adherence, empowerment and blood pressure control. The DANish evaluation of Your heart forecast (DANY) project will be the first to rigorously evaluate effects of YHF in Denmark and to link adherence of hypertensive patients exposed to YHF with the national databases of prescriptions and health services provided.

**Trial registration:**

Clinicaltrials.gov, NCT04058847. Registered on 16 August 2019.

## Background

To improve communication of risk messages, they must be communicated in a way that is understandable and perceived relevant to the patients’ personal life and health. Every individual has their own beliefs, values and understanding, as well as social and psychological background. Therefore, communication tools must be personalised and easy to understand. It is important to bear in mind when designing communication tools, that close to half of the European population has a low degree of health literacy [1], which is associated with poor health outcomes, low self-management and underestimation of personal risk of cardiovascular disease (CVD) [2]*.* Since the risk of CVD is the product of a complex combination of multiple individual risk factors, there is a need for communicating complex information in an easy understandable way. Several tools for calculating and communicating risk of CVD have been developed, but rigorous testing of their communicative effects is sparse.

Adherence is generally low for preventive interventions addressing risk of CVD [3]. Most patients do not feel unwell despite their diagnosis of hypertension or hypercholesterolaemia, and combined with an underestimation of their personal risk, this might be the reason for low adherence to preventive treatment.

In Denmark, general practitioners (GPs) are encouraged by clinical guidelines to conduct an annual blood pressure control assessment for patients with hypertension in order to reach guideline-described levels of blood pressure and to reduce other known risk factors of CVD as well [4–6]. The annual blood pressure control assessment represents a unique and regular opportunity for the GPs to motivate their patients to achieve healthier lifestyles and better adherence to treatment. However, despite all good intentions, 7/10 Danish patients diagnosed with hypertension have inadequately controlled blood pressure [7, 8].

Inadequately controlled blood pressure is associated with an increased risk of CVD and more frequent contact with GPs. Intention to change behaviour is related to the patients’ perception of risk [9]. Consequently, better ways for GPs to communicate risks of CVD and motivate patients for risk-reducing strategies are warranted. Efficient communication requires information to be presented in formats that encourage decision makers, i.e. patients, to automatically extract specific meanings or overall messages [10]. Use of graphical illustrations with distinct features has previously been shown to be an effective and user-friendly tool to communicate risk, especially to people with limited health literacy [2, 10, 11]. A visual decision aid could be a way to improve the blood pressure consultations and to assure that GPs are supported to provide patients with the relevant information. It is suggested that using a decision aid will also systematise the consultation and make it more reproducible [12].

The present study will use the Internet-based risk communication tool Your Heart Forecast (YHF) to evaluate whether it can influence patients’ understanding of risk and adherence to treatment. This will be done paying special attention to patients’ blood pressure, lipid levels, empowerment and health literacy. The rationale behind the tool is to help improve patient–doctor interaction and communication so that the patient can gain an improved understanding of his/her risk of CVD and the modifiability of risk. Patients with hypertension have been chosen as a case study, since high blood pressure is one of the main modifiable risk factors for CVD, as opposed to, for example, age, gender, ethnicity and family history.

YHF is a risk-communication tool, which communicates risks of CVD as personalised, interactive and dynamic visual graphs. Following input from personal health-related data, the GP can, by using YHF, interactively guide the patient through:

- their currently predicted five-year absolute risk of CVD;
- the age at which they would achieve their currently predicted risk of CVD if they had ideal/achievable risk factor control (the heart age);
- their predicted risk of CVD as they get older (the heart forecast);
- their future risk of CVD if their current risk factors are improved.

To succeed in behavioural changes, the patient can be helped in several ways in a setting such as general practice. From the perspectives of the Transtheoretical theory/Stages of Change theory [13] and the Information-Motivation-Behavioural Skills theory [13], the present intervention helps patients in several ways as follows. YHF gives the information of risk of CVD in a new way and at the same time offers a motivation as it immediately and visually shows the number of years you can add before reaching a condition where pharmaceutical treatment is highly recommended. As such, YHF presents the information leg as well as the motivation leg from the Motivation-Behavioural Skills theory, which together is expected to strengthen the patient’s behavioural skills and to add to the likelihood of sustaining a behavioural change.

As it has previously been demonstrated that single event interventions do not have an effect if not followed up, YHF will be introduced in a package deal. YHF will function as the primary intervention and repeated reminders via e-mail will be the secondary component. Patients in the intervention group will, after the consultation with the GP, receive emails every other week, as part of a general health literacy educational program.

The second part of our planned intervention, the reminding e-mail program, aims at the action and maintenance stages regarding the Transtheoretical theory [14]. Every other week, patients in the intervention group will receive an email with a piece of advice on how to live healthier. In the same email, a reminder and a link are inserted, through which the patient can play back the information given at the blood pressure consultation at the GP using YHF. The email program offers the possibility of boosting the patients’ motivation and by that support the ongoing behavioural change.

## Methods/Design

### Aim

The aim is to evaluate the effect of using the YHF visual communication tool on changes in blood pressure and adherence to CVD preventive medications. Further, by means of questionnaires, the aim is to study whether changes in health literacy, adherence, patient empowerment and risk communication are associated with changes in blood pressure, lipid levels and/or lifestyle choices. By means of qualitative interviews, it will be investigated whether the use of the program heightens motivation, increases awareness of risk or creates unwarranted effects such as causing the patients to be anxious.

### Research question

#### Primary research question

- Will the introduction of YHF during an annual blood pressure control consultation lead to improved general health literacy, improved adherence to medication and empowerment after 12 months?

#### Secondary research questions

1. Will blood pressure be reduced among patients in the intervention group compared to the control group after 12 months?
2. Will lower health literacy and/or low empowerment at baseline be associated with higher blood pressure at baseline?
3. Will health literacy and/or empowerment be improved after 6 and/or 12 months among patients enrolled in the intervention group?
4. Will increased health literacy and/or empowerment be associated with healthier lifestyle including diet, exercise and smoking habits after 12 months?
5. Will there be subjective feelings of increased motivation or sickening after introducing the intervention?
6. Will risk of CVD be lower among patients in the intervention group compared to the control group after 12 months?

### Clinical relevance

- Low medication adherence is a significant health, time and cost-consuming challenge for patients with increased blood pressure as well as for their GPs.
- The study is not only relevant for the individual patient, but due to the very high prevalence of increased blood pressure, also of significant societal relevance.
- The study will address this clinically relevant challenge through improved risk communication and efforts to improve health literacy.
- Increased health literacy will most likely lead to fewer visits to the GP, better adherence, lower degree of complications and, thus, higher quality of life for the patient and fewer health costs for society.

### Trial design

This study will use a mixed methods approach with a combination of a randomised controlled trial (RCT) and qualitative semi-structured interviews. The protocol has been developed using the SPIRIT checklist as guideline (see Additional file 1).

Participating general practices will be cluster randomised into two groups, an intervention and a control group. The randomisation will be done using REDCap’s randomisation tool. Two general practices will function as pilot practices and be enrolled as if they were intervention practices. In these two practices, patients will be assessed 3–6 months before the project practices. A subgroup Q, of 5–15 patients from the pilot group, will be selected for qualitative interviews.

GPs in the control group will not be introduced to YHF and will follow their patients in the blood pressure control program as usual.

### Study population

#### Participants

All general practices in the Region of Southern Denmark will receive a postally distributed, written invitation to an information meeting about the RCT. Practices not attending the meetings will, in following inclusion rounds, be offered an introduction to the trial via video meetings. Of those GPs willing to participate, 30 will, with due respect for geographical location and practice type, be representatively selected for participation.

Both incident and prevalent hypertensive patients will be included within an inclusion period of 6–12 months.

### Sample size

Calculation of sample size is based on blood pressure as primary outcome. For a two-sample pooled *t* test of a normal mean difference with a two-sided significance level of 0.05, a sample size of 120 participants per group is required. This is to obtain a power of at least 90%, to detect a difference of 5 mmHg between the means at baseline and after one year. To adjust for expected drop-outs, 30 participants will be added per group and at least 300 patients will be enrolled in the trial. To account for cluster-effects when randomising on practice level, the sample size will be further increased by 10%–15% to reach 340 patients. More participants will be needed for subgroup analyses on sociodemographic and, therefore, the aim is to reach a total of 600 participating patients. The pilot practices will provide information regarding the prevalence of patients with inadequately controlled blood pressure willing to participate, as well as more specific knowledge on the needed number of practices included, making the final sample size calculation uncertain at this point.

### Randomisation

Participating GPs will be randomly divided in to two groups A and B, using the randomisation tool built in to REDCap. They will be given Trial General Practice Numbers (TGN) from A01-A15 (group A) and B01-B15 (group B).

Participants will be given Trial Participant Numbers (TPNs). TPNs will be generated by PREDICT (the software behind YHF) when patients are included and will be given consecutively starting from 0001. The number will be given a prefix A or B depending on which group (intervention or control) the participant’s general practice belongs to. Thus, the sequence of TPNs can vary in prefixes but will consist of unique four-digit numbers (i.e. B0001, B0002, A0003, B0004, A0005, A0006, A0007, etc.). With this method of labelling TPNs and TGNs, there is room for adjustment if the number of participants/GPs ends up being either smaller or greater than the expected 640/32 in total.

Subgroup Q will be 5–15 participants chosen deliberately from the pilot group, to ensure it represents the intervention group for use in the qualitative interviews.

### Inclusion criteria for general practices

1. To ensure comparability to usual care, all the included general practices must use their own routine method to take blood samples, ECG etc., before annual blood pressure control consultations.
  All general practices in Denmark are supposed to follow a national quality guideline and take part in continuing education. This means that the standard routine methods vary only little across the country.
2. To ensure equal quality, all general practices must measure blood pressure using an ambulatory blood pressure device under standard conditions.
3. To ensure comparability to usual care, inclusion and subsequent intervention must be in connection to a planned blood pressure control consultation, as part of the standard blood pressure control program (either at the time of diagnosis or as a planned annually control).

These criteria are controlled when the first author visits the practices in the start-up-faze (see chapter: ‘Practical procedure’).

### Inclusion criteria for patients

1. All patients must understand and read Danish and must be cognitively well functioning (be able to understand the trial information given and thus make a decision on whether to participate on acceptable grounds).
2. The patients must have Internet access, have an email address and read their emails on regular basis (at least once a week).
3. Patients must give informed consent before inclusion.
4. All included patients must be diagnosed with hypertension and participate in blood pressure control consultations with their GP at least once a year.
5. Both patients with known hypertension and those newly discovered are accepted into the trial.
6. Patients must be aged 35–75 years.
7. Men and women are included.
8. Co-morbidity is allowed with a few exceptions (see exclusion criteria).

### Exclusion criteria for patients

1. If the patient, during the trial, no longer fulfils inclusion criteria 1 and/or 2, they are excluded from the trial.
2. If the patient during the trial develops prolonged illness so severe that treatment of hypertension is no longer a priority, he/she will be excluded.
3. Patients with blood pressure > 170/100 are excluded, as these patients should receive intensive blood pressure treatment regardless of their predicted risk of CVD or heart age.
4. Pregnancy.
5. Very high cholesterol (TCL or TCL/HDL ≥ 8).
6. Genetic lipid disorders.
7. If the patient is diabetic and has a complicating kidney disease.
8. Known problems with arteries to the legs defined as:
  - Clinical symptoms of claudication
  - Diminished foot pulses
  - Carotid bruits
  - Radiological evidence of atherosclerotic arterial disease
  - Prior surgery/percutaneous interventions
9. Prior stroke or mini-stroke (TIA).
10. Angina, prior AMI or heart-related operation.

### Practical procedure

**Fig. 1:**
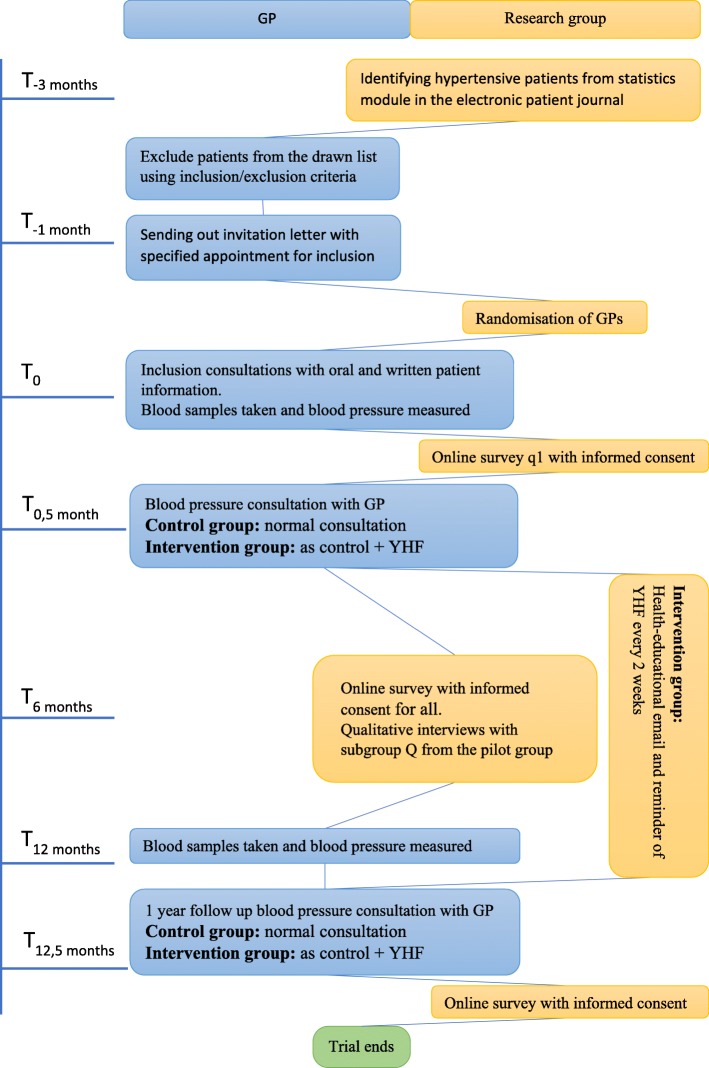
Flow chart

General practices will be actively involved in the RCT as follows (see the trial flow chart in Fig. 1). All participating practices will receive a 1-h introduction to the project in their own clinic, conveyed by the first author (AEJ) and the staff members will at this time also receive relevant documents for further distribution to patients. At the introduction, the first author will assist the GP in drawing a list from the statistics module of their electronic patient journal. The list will show patients with the hypertension diagnoses K85, K86 and K87 and it will be sorted by central personal register number starting with 1 January.

After identifying the list of patients from the statistics module, the practice will review the patients with regards to the inclusion/exclusion criteria. After exclusion, an invitational letter will be sent out to the first 25 patients of those remaining on the list. The invitation will contain a specific appointment for the patient, scheduled for the inclusion consultation with their GP. The invited patients can then opt out or show up at the appointment and receive the oral and written trial participant information (see Additional file 3). The inclusion consultation will be planned before the patients’ expected annual blood pressure consultation and will be planned so they can get blood samples taken at the same time. Thereby patients will not need to go to the practice more times than usual, but the practice will need to conduct an extra GP consultation to give trial information. The participating general practices will be compensated for their extra time spent on consultation with each trial participant and for the extra time spent on reviewing the patient list with regards to inclusion/exclusion criteria.

A few days after the inclusion consultation, the participant will receive an email with a link to a questionnaire (see Additional file 2) that must be completed before the subsequent appointment with the GP. The first page of the questionnaire will be the informed consent form. If the patient answers no to the informed consent, further access to the questionnaire will be closed and the patient will be excluded from the trial.

The GP responsible for the following blood pressure control consultation will ensure that the questionnaire is filled out by the patient. GPs of the intervention group must subsequently guide their patient through YHF on their computer, uploading this individual patient’s data.

With the large number of patients with known hypertension and the compressed method of inclusion, it is expected that GPs will have enough use of the YHF program to maintain the skills and knowledge to use the program, which they will be taught by the research group before start of the project.

The RCT will consist of a 12-month intervention period, except for participants in subgroup Q who will be interviewed six months after enrolment (t_6_) and subsequently excluded. Follow-up for all other patients will happen at the next annual blood pressure control consultation (t_12_) approximately 12 months after registration of baseline data (t_0_).

The intervention group will receive an educational email every two weeks that also includes a reminder of the project and YHF. The first email is sent out approximately two weeks after the initial blood pressure control consultation. The last email is sent out two weeks after the expected one-year follow-up blood pressure control consultation. The emails’ health educational content will reflect available information from the Danish Heart Association’s web page (www.hjerteforeningen.dk).

The GPs are asked to make sure that blood pressures are measured as a standard ambulatory blood pressure just before the annual blood pressure control (t_12_).

### Data collection

Recruitment will take place from summer 2019 until the intended sample size is reached, presumably within 12 months. The RCT is expected to be completed during 2020.

**Fig. 2:**
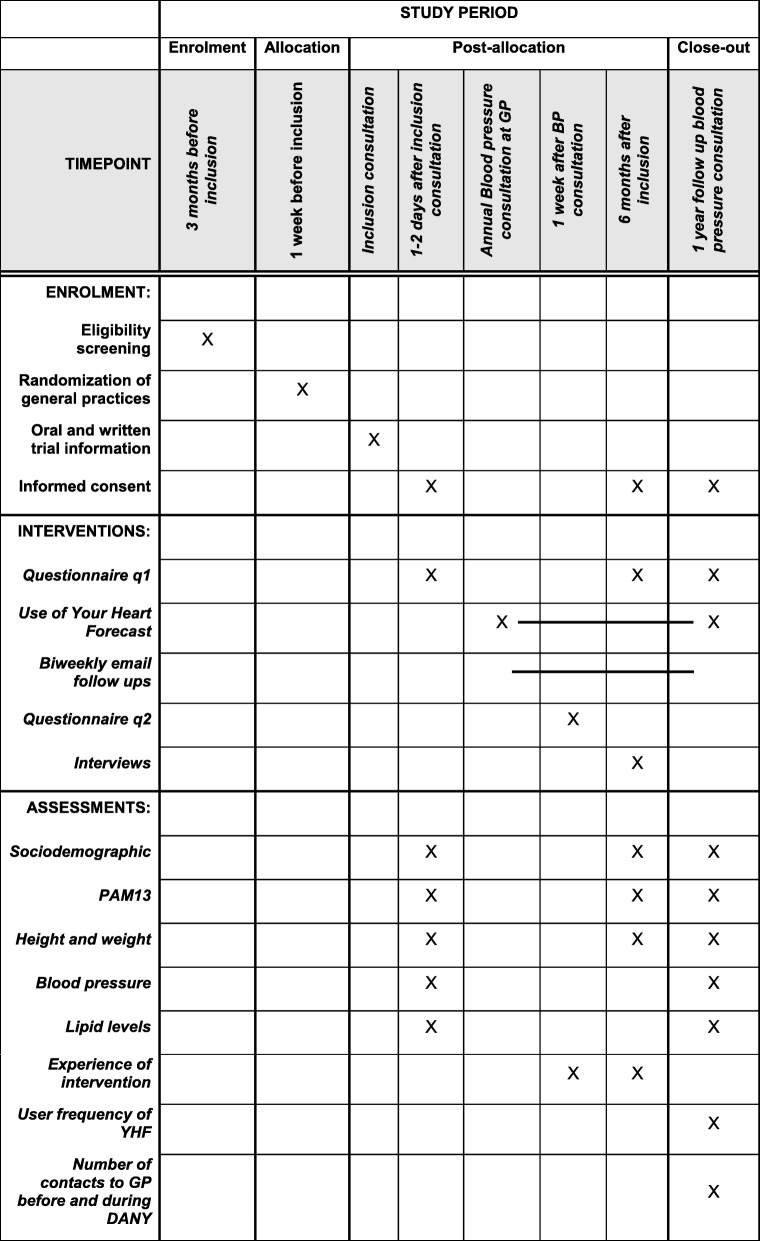
SPIRIT figure

Baseline data will be collected at recruitment (see the SPIRIT figure in Fig.2), via the patient questionnaires and medical records.

The questionnaire (q_1_) will include questions to evaluate socioeconomic and sociodemographic variables, baseline health literacy, risk perception and self-efficacy (PAM-13 [15]), smoking status, co-morbidity and medication.

The second patient questionnaire (q_2_) is very short and will be sent out by email approximately two weeks after the first questionnaire and the informed consent has reached the research group. This questionnaire will focus on whether the patient was surprised about the risk score, if any changes in the medication were made and how the general experience of the YHF is. In this email, the intervention group will be informed that they have been selected to receive an email every second week with information about general health literacy, risks of CVD and advice regarding risk reduction.

Six months after enrolment (t_6_) participants will be asked to answer the first questionnaire (q1) again.

Data regarding blood pressure will be obtained through questionnaires at baseline and after the first annual blood pressure control (t_12_), where blood pressure should also be measured as an ambulatory blood pressure. Data regarding number and content of contacts to the GP will be obtained from the patient’s medical records and the affiliated accounting system including prescription databases for estimating compliance. All telephone consultations, email consultations, clinic consultations and home visits are registered. Contacts will be obtained from three years before and up until two years after the intervention.

Patients in the intervention group will receive their personal profile in the YHF to make it possible for them to access and use the program at home in between the blood pressure consultations at their GP. To gain access to their profile from home, they shall use a personal link sent by email at the end of the blood pressure consultation with their GP. All data entered in YHF will be stored in accordance with Danish law by the software provider, with whom a data management agreement has been made.

Baseline measurements of health literacy, perception of risk and PAM-13 will be used (in conjunction with socioeconomic and sociodemographic variables) to identify subgroups of participants. Qualitative data will be obtained via semi-structured interviews, transcribed and analysed with systematic text condensation. The qualitative interviews will seek to shed light on possible explanations for the hypothesised effects on self-management, lifestyle choices, blood pressure and contact with the GP. If possible, the PhD student will personally make all interviews and transcriptions. In case of time constraints, an assistant will carry out part of the qualitative data collection and transcriptions.

### Outcome measures and statistical analysis

For continuous outcomes, i.e. blood pressure and number of GP visits, linear regression analysis of mean changes from baseline will be carried out. Ordinal regression and multinomial regression will be used for ordered and categorical outcomes, i.e. PAM13 score. Adjustments for baseline values will be made. An intention-to-treat analysis will be applied. Subgroup analyses to identify groups that especially benefit from the intervention will be performed.

Since the authors oversee the project throughout and thereby have access to all data as they are generated, analyses on outcome measures will be carried out by a blinded statistician. Data will be delivered pseudonymised to the statistician.

## Discussion

Despite the variety of CVD risk communication tools that exists, adherence to medications remains low for hypertensive patients. There is a worldwide focus on improving adherence and/or compliance, but often there is a lack of rigorous testing of the tools’ communicative qualities. There is much to be gained by developing adherence-enhancing interventions, maybe even more than by producing new drugs. YHF is a well-used risk communication tool in New Zealand, but it has not been rigorously evaluated there. Since the DANY project will be the first to test YHF in Denmark, it will also be the first to link adherence of hypertensive patients with the YHF and with national databases. It is uncertain to what extent health literacy and empowerment influences patients’ adherence, but both will be important indicators in the DANY project.

### Limitations

It is not possible to control how the GPs act in their own practices. This leaves room for some differences in procedures and information given. This can, however, also be seen as a strength, because the usual care given to the patients by their GP will not be changed. The message drawn from the tool will not be precisely alike for all GPs, but this diversity will reflect real life and that is the goal of the trial – to test whether YHF works in a real-life general practice setting.

Whether patients take the medications they pick up at the pharmacy cannot be controlled. The prescription database should, however, be a reliable reflection of patients’ intake of medicine, since patients presumably take the medications they buy.

The randomisation will be done at GP level instead of patient level. A GP cannot be expected to use the YHF with one patient and not be influenced by the process when the next patient comes. GPs will always intend to give their patients the best treatment they can, but the potential for a spill-over effect is accounted for, by choosing the cluster randomisation process.

The inclusion of GPs will be done by handing out information about the trial and then leaving it up to the GP whether they wish to participate. This might lead to a selection bias, as those willing to participate might have greater interest in hypertension than the GPs not interested in participating. On the other hand, this possible selection bias might give an underestimation of any positive result, if such is to be found, since the participating GPs are possibly already good at treating hypertension.

Use of ambulatory blood pressure measurements will give higher values of blood pressure compared to home blood pressure measurements, but this will only result in a stochastic variance and therefore not influence the outcomes of the trial.

All communication to patients between the consultations is done by email. As reading emails regularly serves as an inclusion criterion, it will potentially exclude some patients with hypertension from participating in the trial. However, given that Denmark has one of the highest levels of Internet coverage of its population, and that all official communication from public institutions to citizens are already sent electronically, it does not seem like a significant challenge.

### Clinical implications/perspectives

It is expected that using YHF will improve health literacy and patient empowerment, increase adherence to medication and lower patients’ blood pressure. Further, socio-demography is expected to have a significant impact on these outcomes. If the introduction of YHF is shown to significantly improve patient adherence or reduce hypertension, it should be implemented on a larger scale. By using YHF, focus is moved towards patients’ total risk of CVD instead of just hypertension.

The wider perspective of the present trial is the generic potential that improved risk communication through a dynamic and interactive communication tool can change not only empowerment, understanding and relevant health outcomes for CVD, but also for other diseases.

## Status of the project

Current protocol is version 2.5, August 2019.

The trial began the pilot phase in March 2019 and the first recruitment consultations for project participants were done in June 2019. Recruitment is planned to be completed in March 2020.

## Supplementary information

**Additional file 1.** The SPIRIT checklist for the protocol.

**Additional file 2.** The first questionnaire the participants receive (q1). It contains the informed consent as the first question. The questionnaire is in Danish.

**Additional file 3.** Written patient information. The material that every patient is handed in hard copy before inclusion. The material is in Danish.

## Data Availability

Access to the final dataset will be limited to the authors’ research groups until publication, after which the datasets analysed will be made available from the corresponding author on reasonable request. Results will be published in peer-reviewed journals as well as at conferences and meeting for participating general practitioners. Authorships will follow the Vancouver declaration.

## Abbreviations

CVD: Cardiovascular disease
GP: General practitioner
RCT: Randomised controlled trial
SDU: University of Southern Denmark
TGN: Trial General Practice Number
TPN: Trial Participant Number
YHF: Your Heart Forecast
