# Supplementary material for: Danish Evaluation of Your Heart Forecast (DANY): study protocol for a cluster randomised controlled trial on an interactive risk-communication tool aimed at improving adherence of patients with high blood pressure
Source: Trials. 2020 Jan 3;21:11. doi: 10.1186/s13063-019-3886-2 (PMC6942322; doi:10.1186/s13063-019-3886-2)
Supplement: Supplementary file 2 — Additional file 2. The first questionnaire the participants receive (q1). It contains the informed consent as the first question. The questionnaire is in Danish. [file 13063_2019_3886_MOESM2_ESM.pdf]

DANY - Spørgeskema q1

Kære [sd03].

Vi er glade for, at du har valgt at deltage i DANY-projektet.

Vi har registreret følgende deltagernummer og lægehus til dig:

Deltagernummer (TPN): [sd02]

Lægehus: [sd01]

Før du begynder på spørgeskemaet, skal du afgive samtykke til deltagelse i projektet.

Det er frivilligt at deltage og hvis du svarer nej til samtykket, vil undersøgelsen blive afsluttet.

Det efterfølgende spørgeskema "q1" er det første af de spørgeskemaer, som vi vil bede dig udfylde i løbet af projektet. Du skal kun besvare dette ene spørgeskema i denne omgang.

Spørgeskemaet er delt i 5 dele (A-E) og indeholder i alt 56 spørgsmål.

Del A er 15 indledende spørgsmål om dig og dit blodtryk.

Del B er 13 spørgsmål om din personlige deltagelse i håndteringen af din sygdom.

Del C er 9 spørgsmål om din behandling.

Del D er 9 spørgsmål om risikofaktorer for sygdom.

Del E er 10 spørgsmål om din baggrund.

Du kan vælge at besvare hele spørgeskemaet på en gang, eller du kan besvare nogle af spørgsmålene, gemme og vende tilbage på et senere tidspunkt.

Det er vigtigt at du har udfyldt hele spørgeskemaet før din næste aftale hos lægen.

God fornøjelse og igen, tak for hjælpen.

Venlig hilsen

Anders Elkær Jensen

Læge, Ph.d.-studerende

Syddansk Universitet

Forskningsenheden for Almen Praksis

J.B. Winsløws Vej 9A

5000 Odense C

Kontor: 3.29, 2. sal

E-mail: anejensen@health.sdu.dk

T +45 6550 4794 / M +45 2982 8718

Når du har læst ovenstående tekst klikker du på "næste side" for at komme til samtykket.

Informeret samtykke til deltagelse i et sundhedsvidenskabeligt forskningsprojekt.

Informeret samtykke.

Forskningsprojektets titel:
Evaluering af "Dit hjertes fremtid".
Et studie blandt danske alment praktiserende læger og patienter i kontrolprogrammet for forhøjet blodtryk.

Original titel:
Evaluation of Your Heart Forecast (DANY).
A study among Danish general practitioners and patients in the blood pressure control program.

Projekt ID ved Videnskabsetisk komité: S-20170206

Erklæring fra forsøgspersonen:
Jeg har fået skriftlig og mundtlig information og jeg ved nok om formål, metode, fordele og ulemper til at sige ja til at deltage.

Jeg ved, at det er frivilligt at deltage, og at jeg altid kan trække mit samtykke tilbage uden at miste mine nuværende eller fremtidige rettigheder til behandling.

Jeg giver samtykke til at deltage i forskningsprojektet og til at der må indhentes oplysninger fra min patientjournal.
Oplysninger må hentes for perioden 3 år forud for inklusion i forsøget og til 2 år efter afslutning af forsøget.
Jeg har fået en kopi af dette samtykkeark samt en kopi af den skriftlige information om projektet til eget brug.

Forsøgsdeltagerens navn: [sd03]

CPR-nummer: [sd04]

Deltagernummer (TPN): [sd02]

(Kopi af dette samtykkeark samt en kopi af den skriftlige information om projektet kan downloades via nedenstående link)

[Attachment: "Deltagerinfo samlet 2.1 Marts 2019.pdf"]

Jeg giver mit samtykke som ovenfor beskrevet

○ Ja
○ Nej

Underskrift	__________________________________
Ønsker du at blive informeret om forskningsprojektets resultat samt eventuelle konsekvenser for dig?:	◯ Ja<br>◯ Nej
Hvilken enhed bruger du til at tilgå din e-mail?	◯ PC (styresystem fx Windows eller Linux)<br>◯ Apple-computer (styresystem macOS)<br>◯ Tablet med fx Android eller windows styresystem<br>◯ Tablet med iOS styresystem (fx iPad)<br>◯ Telefon med fx Android eller windows styresystem<br>◯ Telefon med iOS styresystem (fx iPhone)<br>◯ Andet

Del A
Indledning

1	Er dit deltagernummer (TPN): [sd02] og din læge: [sd01]?	◯ Ja<br>◯ Nej<br>(Det er vigtigt for projektet at alle dine besvarelser gemmes under samme deltagernummer (TPN). Hvis du svarer nej, afsluttes spørgeskemaet og du bedes kontakte projektleder Anders Elkær Jensen på anejensen@health.sdu.dk for at få rettet dit deltagernummer.)
2	Hvad er dit systoliske blodtryk (målt i forbindelse med at du fik taget blodprøver)?	__________________________<br>(Et blodtryk skrives fx som 120/80, hvor det største tal hedder det systoliske blodtryk.)
3	Hvad er dit diastoliske blodtryk (målt i forbindelse med at du fik taget blodprøver)?	__________________________<br>(Et blodtryk skrives fx som 120/80, hvor det mindste tal hedder det diastoliske blodtryk.)
4	Hvilken dato fik du målt dit blodtryk (målt i forbindelse med at du fik taget blodprøver)?	__________________________
5	Hvor høj er du (cm)?	__________________________
6	Hvor meget vejer du (kg)?	__________________________
	BMI	__________________________<br>(Resultatet vil altid blive angivet med rød skrift. Det er ingen vurdering af om dit BMI er for højt, for lavt eller tilpas.)
7	Hvad er din livvidde (cm)?	__________________________<br>(Livvidden måles i det bløde område over hoftekammen og er forskellig fra hoftemålet som måles på det bredeste sted omkring balder/lår.)
8	Hvad er dit køn?	◯ Kvinde<br>◯ Mand
9	Hvad er din alder (angivet i hele år)?	__________________________
10	Hvornår fik du diagnosen forhøjet blodtryk?	__________________________<br>(Hvis du ikke kender den nøjagtige dato, så sæt dato til den 1. i den måned du fik diagnosen. Hvis du ikke kan huske måneden, så sæt datoen til 1/1 det år hvor du fik diagnosen.)
11	Har du aftalt med lægen hvad behandlingsmålet for dit blodtryk er?	◯ Ja<br>◯ Nej

12 Hvis ja, hvad er målet da for dit systoliske blodtryk?

____
(Et blodtryk skrives fx som 120/80, hvor det største tal hedder det systoliske blodtryk.)

13 Hvis ja, hvad er målet da for dit diastoliske blodtryk?

____
(Et blodtryk skrives fx som 120/80, hvor det mindste tal hedder det diastoliske blodtryk.)

14 Hvor alvorlig mener du at din blodtrykssygdom er?

- ◯ Meget alvorlig
- ◯ Alvorlig
- ◯ Ikke så alvorlig
- ◯ Slet ikke alvorlig

15 Måler du dit blodtryk hjemme?

- ◯ Ja
- ◯ Nej

Del B

Spørgsmål om personlig involvering.

Hvor enig eller uenig er du i følgende udsagn?

		Meget enig	Enig	Uenig	Meget uenig	Ved ikke
16	Når alt kommer til alt, er det mig, der har ansvaret for at tage vare på mit helbred.	○	○	○	○	○
17	Min egen aktive rolle i behandlingen er den vigtigste faktor for mit helbred og evne til at fungere.	○	○	○	○	○
18	Jeg er sikker på, at jeg selv kan gøre noget for at forebygge eller mindske nogle af de symptomer eller problemer, der er forbundet med forhøjet blodtryk.	○	○	○	○	○
19	Jeg ved, hvordan de forskellige slags lægeordinerede medicin jeg får, virker.	○	○	○	○	○
20	Jeg er sikker på, at jeg selv kan afgøre, hvornår jeg har brug for at søge behandling og hvornår jeg selv kan klare det.	○	○	○	○	○
21	Jeg er sikker på, at jeg kan fortælle lægen om mine bekymringer, selv om lægen ikke spørger.	○	○	○	○	○
22	Jeg er sikker på, at jeg derhjemme kan følge den anbefalede behandling.	○	○	○	○	○
23	Jeg forstår, hvad forhøjet blodtryk indebærer og hvad der er årsag til det.	○	○	○	○	○
24	Jeg kender de forskellige behandlingsmuligheder, der findes for forhøjet blodtryk.	○	○	○	○	○
25	Jeg har været i stand til at fastholde de ændringer i livsstil, jeg har foretaget af hensyn til mit helbred.	○	○	○	○	○
26	Jeg ved, hvordan jeg skal forhindre yderligere problemer med mit helbred.	○	○	○	○	○
27						

	Jeg er sikker på, at jeg kan finde en løsning, når der opstår en ny situation eller nye problemer med mit helbred.	○	○	○	○	○
28	Jeg er sikker på, at jeg, selv i stressede perioder, kan fastholde ændringer i min livstil fx kost og motion.	○	○	○	○	○

Del C
Spørgsmål om din behandling

29	Er du, eller har du været i behandling med lægeordineret medicin mod forhøjet blodtryk?	◯ Ja<br>◯ Nej
30	Hvor mange forskellige lægemidler tager du for dit forhøjede blodtryk? (kun medicin på recept)	______________________<br>(Skriv antal forskellige lægemidler og medtag kun lægemidler du får pga dit forhøjede blodtryk)
31	Anvender du doseringsæske til din medicin?	◯ Ja<br>◯ Nej
32	Har lægen eller sygeplejersken spurgt dig, om du af og til glemmer at tage medicinen eller på anden vis ikke tager den korrekt?	◯ Ja<br>◯ Nej
33	Har du oplevet noget, du opfatter som bivirkninger til blodtryks-medicinen?	◯ Ja<br>◯ Nej
34	Hvis ja, har du talt med lægen eller sygeplejersken om det?	◯ Ja<br>◯ Nej
35	Har din læge spurgt dig, om du har haft bivirkninger af din blodtryksmedicin?	◯ Ja, ved hver konsultation<br>◯ Ja, ofte<br>◯ Kun sjældent<br>◯ Nej, aldrig
36	Hvem har udført dine blodtrykskontroller i det sidste år? Sæt gerne flere krydser.	☐ Din egen læge<br>☐ Andre læger i din praksis<br>☐ Sygeplejerske i din praksis<br>☐ Andet personale i din praksis<br>☐ Sygehus-ambulatorium<br>☐ Andre, fx praktiserende speciallæge
37	Er det indenfor den sidste uge hændt, at du har glemt at tage din medicin mod dit forhøjede blodtryk?	◯ Ja<br>◯ Nej

Del D
Spørgsmål om risikofaktorer

38 Ryger du?

- ◯ Ja, tobak
- ◯ Ja, E-cigaretter
- ◯ Nej - tidligere ryger
- ◯ Nej - aldrig

39 Hvis ja, hvor mange cigaretter svarer det til at du ryger dagligt?

____

40 Tidligere ryger, hvornår stoppede du?

____

(Hvis du ikke kender den nøjagtige dato, så sæt dato til den 1. i den måned hvor du stoppede. Hvis du ikke kan huske måneden, så sæt datoen til 1/1 det år hvor du stoppede med at ryge.)

41 Hvor mange genstande alkohol (øl, vin, spiritus) drikker du sædvanligvis om ugen?

1 alm. pilsner = 1 genstand
1 flaske rød-/hvidvin = 6 glas (genstande)
1 flaske hedvin = 10 glas (genstande)
1 flaske spiritus = 20 genstande

- ◯ 0
- ◯ 1-7
- ◯ 8-20
- ◯ 21-50
- ◯ mere end 50

42 Har du nu, eller har du tidligere haft forhøjede kolesteroltal?

- ◯ Ja
- ◯ Nej

43 Vejer du for meget?

- ◯ Ja
- ◯ Nej

44 Har dine forældre eller søskende haft forhøjet blodtryk?

- ◯ Ja
- ◯ Nej

45 Har du, nu eller tidligere, haft én eller flere af følgende sygdomme? (sæt evt. flere krydser)

- ☐ Allergi
- ☐ Blodprop i hjertet
- ☐ Dårligt hjerte
- ☐ Forhøjet stofskifte
- ☐ Kræft
- ☐ Leversygdom
- ☐ Lungesygdom (ikke almindelig lungebetændelse)
- ☐ Nyreskade
- ☐ Slagtilfælde (blodprop i hjernen eller hjerneblødning)
- ☐ Slidgigt
- ☐ Sukkersyge
- ☐ Synssvækkelse og/eller anden øjensygdom
- ☐ Åreforkalkning i benene
- ☐ Ingen af ovennævnte
- ☐ Andet

46 Hvilke andre sygdomme har du nu eller har du tidligere haft?

____

Del E
Spørgsmål om din baggrund

47	Hvor ofte har du det sidste år haft kontakt med din praktiserende læge (fx fremmøde, hjemmebesøg, tlf, email)?	◯ Mindre end 5 gange<br>◯ 5-20 gange<br>◯ Mere end 20 gange
48	Har disse kontakter drejet sig om din blodtrykssygdom?	◯ Slet ikke<br>◯ I nogen grad<br>◯ Ofte<br>◯ I meget høj grad
49	Hvad er din ægteskabelige status?	◯ Gift eller samlevende<br>◯ Enlig (skilt eller afbrudt samlivsforhold)<br>◯ Enlig (enke, enkemand)<br>◯ Enlig (ikke tidligere gift eller samlevende)
50	Har du børn (uanset alder)?	◯ Ja<br>◯ Nej
51	Hvor gamle er dine børn/dit barn?	______________________________<br>(del aldrene med , som fx 5år, 12år, 58år, 43år)
52	Hvilken uddannelse har du gennemført (sæt kun 1 kryds)?	◯ Folkeskolen<br>◯ Ungdomsuddannelse (fx erhvervsuddannelse, gymnasium eller social- og sundhedsuddannelse)<br>◯ Kort eller mellemlang videregående uddannelse (1-4 år)<br>◯ Lang videregående uddannelse (5 år eller mere)
53	Hvordan er du beskæftiget?	◯ I arbejde<br>◯ Arbejdsløs<br>◯ Førtidspensionist<br>◯ Folkepensionist<br>◯ Hjemmegående<br>◯ I arbejde, men på orlov<br>◯ Sygemeldt<br>◯ Under uddannelse
54	Hvordan vurderer du din nuværende helbredstilstand i almindelighed?	◯ Fremragende<br>◯ Vældig god<br>◯ God<br>◯ Mindre god<br>◯ Dårlig

55 Hvilke af følgende sygdomme mener du, kan forårsages af forhøjet blodtryk (sæt evt. flere krydser)?

- ☐ Allergi
- ☐ Blodprop i hjertet
- ☐ Dårligt hjerte
- ☐ Forhøjet stofskifte
- ☐ Kræft
- ☐ Leversygdom
- ☐ Lungesygdom (ikke almindelig lungebetændelse)
- ☐ Nyreskade
- ☐ Slagtilfælde (blodprop i hjernen eller hjerneblødning)
- ☐ Slidgigt
- ☐ Sukkersyge
- ☐ Synssvækkelse og/eller anden øjensygdom
- ☐ Åreforkalkning i benene
- ☐ Ingen af ovennævnte
- ☐ Andet

56 Hvilke andre sygdomme mener du, kan forårsages af forhøjet blodtryk?

____

Mange tak, du har nu færdiggjort spørgeskema q1.
Afslut ved at trykke på "indsend".
