# Supplementary material for: Danish Evaluation of Your Heart Forecast (DANY): study protocol for a cluster randomised controlled trial on an interactive risk-communication tool aimed at improving adherence of patients with high blood pressure
Source: Trials. 2020 Jan 3;21:11. doi: 10.1186/s13063-019-3886-2 (PMC6942322; doi:10.1186/s13063-019-3886-2)
Supplement: Supplementary file 3 — Additional file 3. Written patient information. The material that every patient is handed in hard copy before inclusion. The material is in Danish. [file 13063_2019_3886_MOESM3_ESM.pdf]

”DANY-PROJEKTET”

Deltagermateriale

Forskningsprojektet

Dansk Evaluering af ”Dit hjertes fremtid”.
Et studie blandt danske alment praktiserende læger og patienter i kontrolprogrammet for forhøjet blodtryk

Læge Anders Elkær Jensen
anejensen@health.sdu.dk

Indledning

Vi vil spørge, om du vil deltage i et videnskabeligt forsøg, der udføres af Forskningsenheden for Almen Praksis ved Syddansk Universitet i samarbejde med sundhedsvæsnet i Region Syddanmark.

Før du beslutter, om du vil deltage i forsøget, skal du fuldt ud forstå, hvad forsøget går ud på, og hvorfor vi gennemfører forsøget. Vi vil derfor bede dig om at læse denne deltagerinformation grundigt.

Når du har besluttet dig for om du vil deltage i forsøget, vil vi bede dig om at udfylde et spørgeskema online.
Her vil du have mulighed for at angive om du ønsker at deltage eller ej.
Du vil få tilsendt et link til spørgeskemaet på den e-mail du har oplyst til lægehuset.
Husk, at du har ret til betænkningstid, før du beslutter, om du vil deltage i forsøget.
Det er frivilligt at deltage i forsøget. Du kan når som helst og uden at give en grund trække dit samtykke tilbage.

På de næste sider har vi samlet relevant informationsmateriale til dig. Du skal opfatte denne skriftlige information som tillæg til den mundtlige information du har fået i dit lægehus.
Vi håber, at du med denne information har fået tilstrækkeligt indblik i, hvad det vil sige at deltage i forsøget og at du føler dig rustet til at tage beslutningen om din eventuelle deltagelse.

Alle spørgeskemaer i projektet udsendes fra en regional e-mailadresse (OUH.ODE.OP539@rsyd.dk), men du kan ikke svare os på denne adresse.
Hvis du vil vide mere om forsøget, eller har spørgsmål undervejs i forløbet, så er du meget velkommen til at kontakte mig på anejensen@health.sdu.dk.

Med venlig hilsen

Anders Elkær Jensen
Læge, Ph.d.-studerende

Syddansk Universitet
Forskningsenheden for Almen Praksis
J.B. Winsløws Vej 9A
5000 Odense C

E-mail: anejensen@health.sdu.dk
www.sdu.dk

T +45 6550 4794

SDU

Målte værdier:

CPR-nummer:	_______________-________
Dato (dd-mm-åååå):	
Højde:	cm
Vægt:	kg
Livvidde:	cm
Systolisk blodtryk:	mmHg
Diastolisk blodtryk:	mmHg

Deltagerinformation

Deltagerinformation om deltagelse i det videnskabelige projekt:

Dansk Evaluering af "Dit hjertes fremtid" (DANY).
Et studie blandt danske alment praktiserende læger og patienter i kontrolprogrammet for forhøjet blodtryk.

Formål med projektet
Det er svært at få patienter til at følge forebyggende behandlinger fordi de typisk ikke oplever symptomer på den tilstand, som ønskes behandlet. Eksempler er højt kolesterol og højt blodtryk, som ikke i sig selv giver symptomer.
I dette studie vil vi afprøve et internetbaseret program, der fortæller om risikoen for hjerte-kar-sygdom hos patienter med forhøjet blodtryk. Formålet er primært at øge den enkelte patients viden samt, at få dem til i højere grad at efterleve de aftaler de har med deres læge (bedre deres compliance) og derved opnå bedre behandling.
Cirka 7 ud af 10 danskere diagnosticeret med forhøjet blodtryk har ikke fået det behandlet godt nok og en del opnår derfor ikke den bedst mulige gevinst.
De årlige blodtrykskontroller i almen praksis er en lejlighed til at motivere patienterne til en sundere livsstil samt at efterleve den aftalte behandlingsplan bedre. Der er dog behov for nye pædagogiske værktøjer til at understøtte lægens indsats.

Your Heart Forecast (*Dit hjertes fremtid*) er et program, som synliggør og formidler risiko for hjerte-kar-sygdom og gevinst med medicin og livsstilsomlægninger. Programmet kan bruges på computer, men ikke på smartphone. *Dit hjertes fremtid* lader dig som patient deltage i beslutningstagningen og hjælper med at motivere dig til en sundere livsstil. Ved hjælp af dette værktøj håber vi at understøtte lægens pædagogiske indsats og derved opnå forbedring af patienternes viden om og forståelse af risiko for hjerte-kar-sygdom og mulige gevinster af medicin og livsstilsændringer. Programmet guider dig (patienten) gennem din risiko for hjerte-kar-sygdom. Først vises den aktuelle risiko, derefter en sammenligning med risikoen ved bedst mulig behandling (hjertealderen), så din risiko når du bliver ældre og til sidst den fremtidige risiko hvis du ændrer på dine risikofaktorer.

Plan for projektet
Projektet skal starte i 2019 og løbe indtil sidste patient har fået foretaget sidste kontrol hos lægen (forventet sommer 2021). Der skal bruges ca. 600 patienter som bliver fundet gennem 30 praktiserende læger. Lægerne fordeles tilfældigt i grupperne A og B og patienter tilhører automatisk den gruppe deres læge er blevet udvalgt til. Forsøgsdeltagerne bliver inkluderet i forsøget når de får taget blodprøver og målt blodtryk forud for en planlagt blodtryks-konsultation hos deres læge. Forsøgsdeltagerens rolle i projektet afsluttes når den første årlige blodtrykskontrol er gennemført, altså 1 år efter start. I forbindelse med forsøget trækkes oplysninger fra deltagerens besøg hos lægen 3 år forud. Der trækkes oplysninger om bl.a. antal af kontakter, arten af kontakter og henvendelsesårsager for bedre at kunne vurdere effekten af forsøget. Forsøgsdeltageren vil blive bedt om at besvare et online spørgeskema 3-4 gange undervejs i forsøget; ved opstart (t_0), inden for den første måned (t_0), efter 6 måneder (t_6) og efter 12 måneder ved afslutning af forsøget (t_{12}). Halvvejs i forsøget (t_6) vil der blive lavet interview med 5-15 tilfældigt udvalgte deltagere (Q) fra interventionsgruppen.

Interventionsgruppen skal bruge *Dit hjertes fremtid* og vil modtage regelmæssige e-mails (ca. hver 2. uge) med generelle sundhedsdannende oplysninger samt et link til *Dit hjertes fremtid*. Med dette link er det muligt for deltageren at gense sin personlige risikoprofil.

Bivirkninger, risici, komplikationer og ulemper
Der er ingen bivirkninger ved forsøget, da der ikke bruges medicin ud over den i forvejen udskrevne behandling fra din egen læge.

DANY Studiedesign	t_0	t_6	t_{12}	$t_{24} + t_{36}$
Gr A, intervention	$x + q_1 + q_2$	$q_1 + Q$	$x + q_1$	x
Gr B, kontrol standardbehandling	$x + q_1$	q_1	$x + q_1$	x
x = Blodtrykskontrol hos egen læge<br>Q = kvalitative interviews med undergruppe Q<br>q_1 = online spørgeskema omhandlende flere perspektiver af sundhed<br>q_2 = online spørgeskema omhandlende første oplevelse med YHF				

Nytte ved projektet
Ved at deltage i dette forsøg vil du forhåbentlig opnå en bedre behandling af dit blodtryk. Der ud over kan studiet være til nytte for sundhedsvæsnet generelt.
Megen ny teknologi indføres kritikløst i sundhedsvæsnet uden forudgående relevant evaluering. I dette studie vil vi afprøve et nyt værktøj. Resultaterne vil kunne give anledning til dels om et sådant værktøj generelt bør tages i brug, dels om justeringer bør finde sted og endelig om det bør tilbydes til alle eller kun til udvalgte grupper af patienter.

Deltagelse og udelukkelse fra eller afbrydelse af projekt
Krav for deltagende lægehuse:

1. For at sikre sammenlignelighed med vanlig praksis skal alle deltagende lægehuse tage blodprøver, hjertekardiogram osv. med deres vanlige metoder forud for blodtrykskonsultationen hos lægen.
2. For at sikre kvalitet og reproducerbarhed, skal alle deltagende lægehuse måle forsøgsdeltagernes blodtryk i klinikken og under standardforhold.
3. For at sikre sammenlignelighed med vanlig praksis, skal opstart i forsøget og efterfølgende introduktion til *Dit hjertes fremtid,* ske i forbindelse med en blodtrykskontrol planlagt som led i standardprogrammet for blodtrykskontrol (enten ved diagnosticering eller ved planlagt årskontrol).

Inklusionskrav for deltagende patienter:

1. Alle forsøgsdeltagere skal kunne forstå og læse dansk og skal være kognitivt velfungerende.
2. Alle forsøgsdeltagere skal have internetadgang, have en e-mailadresse og læse deres e-mail regelmæssigt (mindst 1 gang ugentligt).
3. Forsøgsdeltagere skal afgive informeret samtykke forud for deltagelse i forsøget.
4. Alle forsøgsdeltagere skal være diagnosticeret med forhøjet blodtryk.
5. Alle deltagere skal have haft deres sidste blodtrykskontrol hos deres læge indenfor de sidste 6 til 24 måneder forud for inklusionen.
6. Både patienter med nydiagnosticeret og med kendt forhøjet blodtryk inkluderes i forsøget.
7. Alder mellem 35 og 75 år (begge inklusive).

8. Begge køn kan deltage I forsøget.
9. Forsøgspersoner med andre sygdomme (komorbiditet) i tillæg til forhøjet blodtryk accepteres med få undtagelser (se eksklusionskriterier).

Eksklusionskriterier for patienter:

1. Hvis der er mere end 3 måneder til patientens næste planlagte årlige blodtrykskontrol.
2. Hvis patienten i løbet af forsøget ikke længere opfylder inklusionskravene 1 og/eller 2.
3. Hvis patienten under forsøget udvikler længerevarende alvorlig sygdom der gør, at behandlingen af det forhøjede blodtryk ikke længere prioriteres.
4. Kraftigt forhøjet blodtryk på 170/100 eller mere, idet der da skal ske intensiveret behandling og patienten ikke længere kan følge de årlige blodtrykskontroller.
5. Hvis patienten er/bliver gravid.
6. Meget forhøjet kolesteroltal (Total-kolesterol eller TCL/HDL-ratio på 8 eller over).
7. Hvis patienten har en diagnosticeret genetisk betinget lipidforstyrrelse.
8. Hvis patienten har diabetes OG komplicerende nyresygdom.
9. Ved problemer med arterierne til benene defineret som:
    a. Kliniske symptomer på claudicatio (vindueskigger-syndrom).
    b. Formindskede fodpulse.
    c. Mislyde over carotiderne (halspulsårer)
    d. Radiologiske beviser
    e. Tidligere operation/perkutan intervention
10. Tidligere hjerneblødning, blodprop i hjernen eller mini-blodprop i hjernen (TIA/TCI).
11. Hjertesmerter (angina), tidligere blodprop I hjertet (AKS/AMI) eller hjerterelaterede operationer.

Oplysninger om økonomiske forhold

Initiativtager til projektet er Forskningsenheden for Almen Praksis ved Syddansk Universitet.
Projektet finansieres ved 1 års stipendium fra SDU, 1 års stipendium fra Region Syddanmark og øvrig finansiering lægges af Forskningsenheden via bevillinger.
Der ydes ikke støtte til projektet fra private firmaer ud over softwareudvikleren ENIGMA, der giver gratis adgang (licenser) til programmet i projektperioden. Der betales for al anden service ifm. brug af programmet.
De forsøgsansvarlige er alle ansatte ved Forskningsenheden for Almen Praksis ved Syddansk Universitet.

Der ydes ikke økonomisk vederlag til patienter for deltagelse i projektet.
Deltagende læger i projektet honoreres for den ekstra tid de bruger på patientkonsultationer ift. den vanlige behandling.

Adgang til forsøgsresultater

Projektets overordnede datasikkerhedsansvarlige er SDUs databeskyttelsesrådgiver som ved generelle spørgsmål kan kontaktes på dpo@sdu.dk. Ved spørgsmål specifikt til DANY-projektet skal den projektansvarlige kontaktes.
Projektets resultater søges udgivet i internationalt anerkendte tidsskrifter.
Det forventes at forsøget afsluttes i 2021 og at (del-)resultater vil blive udgivet i 2020-22.

Projektansvarlige:

Læge og ph.d.-studerende Ander Elkær Jensen
Hovedvejleder Professor Jesper Bo Nielsen

Informeret samtykke

Informeret samtykke til deltagelse i et sundhedsvidenskabeligt forskningsprojekt.

Forskningsprojektets titel:
Dansk Evaluering af "Dit hjertes fremtid".
Et studie blandt danske alment praktiserende læger og patienter i kontrolprogrammet for forhøjet blodtryk.

Original titel:
Evaluation of Your Heart Forecast.
A study among Danish general practitioners and patients in the blood pressure control program.

Projekt ID ved Videnskabsetisk komité: S-20170206

Erklæring fra forsøgspersonen:
Jeg har fået skriftlig og mundtlig information og jeg ved nok om formål, metode, fordele og ulemper til at sige ja til at deltage.

Jeg ved, at det er frivilligt at deltage, og at jeg altid kan trække mit samtykke tilbage uden at miste mine nuværende eller fremtidige rettigheder til behandling.

Jeg giver samtykke til at deltage i forskningsprojektet og til at der må indhentes oplysninger fra min patientjournal. Oplysninger må hentes for perioden 3 år forud for inklusion i forsøget og til 2 år efter afslutning af forsøget.
Jeg har fået en kopi af dette samtykkeark samt en kopi af den skriftlige information om projektet til eget brug.

Forsøgspersonens navn: ____

CPR-nummer:_______________ - ________

Deltagernummer (TPN):______________

Dato: ________________ Underskrift: ____

Ønsker du at blive informeret om forskningsprojektets resultat samt eventuelle konsekvenser for dig?:

Ja _____ (sæt x) Nej _____ (sæt x)

KOPI

Revideret august 2014

Før du beslutter dig

Om at være forsøgsperson
i sundhedsvidenskabelige forsøg

NVK

NATIONAL
VIDENSKABSETISK
KOMITÉ

Hvis du overvejer at deltage i forsøg

For at få ny viden om sygdomme og blive bedre til at behandle dem, er det vigtigt at forske på mennesker. Kun gennem forskning kan man blive bedre til at forebygge, diagnosticere og ikke mindst at behandle sygdomme.

Du er muligvis blevet spurgt, om du vil deltage i et forsøg – eller du er interesseret i at få noget at vide om, hvad det vil sige at være forsøgsperson.

Hvis du er blevet spurgt, om du vil deltage – fx af din egen læge eller af hospitalet, kan forsøget bl.a. dreje sig om en ny type medicin, supplerende undersøgelser eller en ny operationsmetode. Hvis du er rask og selv overvejer at henvende dig, evt. på baggrund af en annonce, kan forsøget fx handle om kroppens funktioner, livsstil eller forebyggelse af sygdomme.

Nogle ønsker at deltage i et forsøg i forventning om at opnå en bedring af deres sygdom, andre ønsker at deltage for at hjælpe forskningen og dermed fremtidige patienter. Uanset hvad du ønsker, kan denne pjece oplyse dig om de videnskabsetiske komiteer, som skal godkende alle sundhedsvidenskabelige forsøg i Danmark, hvad det vil sige at være forsøgs-person i sundhedsvidenskabelige forsøg og om dine rettigheder som forsøgsperson.

Pjecen oplyser også om forskellige typer af forskning, og om den information, du har krav på at få, inden du tager stilling.

De videnskabsetiske komiteer

De videnskabsetiske komiteer består af både fagfolk (sundhedsvidenskabeligt uddannede) og lægfolk – med et flertal af lægfolk. Komiteerne skal varetage forsøgspersonernes interesser. For selv om det er vigtigt at sikre sundhedsvæsenet ny viden, kommer hensynet til forsøgspersonerne altid først. Komiteerne skal derfor godkende alle sundhedsvidenskabelige forsøg i Danmark.

Før et forsøg kan godkendes, vurderer den videnskabsetiske komité både risikoen for forsøgspersonerne og nytten ved forsøget. Hvis et forsøg på nogen måde er uforsvarligt at gennemføre, bliver det ikke godkendt. Komiteen kan også afvise et forsøg, hvis den mener, at informationen til dig ikke er god nok. Den videnskabsetiske komité sikrer desuden, at der er en erstatnings- eller godtgørelsesordning, hvis en forsøgsperson skulle lide skade på grund af forsøget.

Om at være forsøgsperson

At være forsøgsperson vil sige, at man frivilligt deltager i et sundhedsvidenskabeligt forsøg. Forsøget udføres, fordi forskere mener, at den nye behandling måske er bedre eller mindst lige så god som den hidtidige standardbehandling. Men de ved det ikke med sikkerhed, og det er derfor, man laver forsøget.

Lovgivningen fastsætter, hvordan og på hvilke betingelser et forsøg må gennemføres. Lovgivningen er til for at sikre forsøgspersonens ret-tigheder, som gælder, uanset om deltageren er patient eller en rask person. Hvis du er patient og beslutter ikke at deltage, har du naturligvis altid sikkerhed for, at du alligevel vil modtage den bedste, kendte behandling (standardbehandlingen).

En del forsøg sammenligner standardbehandlingen med en ny forsøgsbehandling, som forskerne mener, kan være bedre. Det gøres ved at fordele deltagerne tilfældigt i to grupper – én, der får standardbehandlingen og én, der får forsøgsbehandlingen. Fordelingen kaldes randomisering og foretages for, at de to behandlinger kan sammenlignes bedst muligt.

Nogle kan føle utryghed ved den tilfældige fordeling af behandlinger. Men det er vigtigt at understrege, at de, der får forsøgsbehandlingen, muligvis får en bedre behandling end de, der får standardbehandlingen. De, der får standardbehandlingen, har til gengæld sikkerhed for at få en velafprøvet behandling. Hvis det undervejs i forsøget viser sig, at forsøgsbehandlingen er dårligere end standardbehandlingen, bliver forsøget afbrudt og alle forsøgspersoner vil derefter modtage standardbehandlingen.

Nogle forsøg tager flere år, og når et forsøg er afsluttet, gør forskerne resultaterne op. Herefter kan de vurdere, om forsøgsbehandlingen har vist sig bedre end standardbehandlingen, eller de kan bruge den nye viden til at gøre fremtidige behandlinger bedre.

Forsøgspersoners rettigheder

- **Din deltagelse i forsøget er helt frivillig,** og inden du eventuelt underskriver samtykkeerklæringen, skal du have både skriftlig og mundtlig information om forsøget.
- **Du kan til enhver tid mundtligt, skriftligt eller ved anden klar tilkendegivelse trække dit samtykke til at deltage tilbage og dermed træde ud af forsøget.** Selv om du trækker dit samtykke tilbage, påvirker dette ikke din ret til nuværende eller fremtidig behandling eller andre af dine rettigheder.
- **Du har ret til at tage et familiemedlem, en ven eller en bekendt med,** som bisidder til informationssamtalen.
- **Du har ret til betænkningstid,** før du giver tilsagn om at deltage.
- **Oplysninger om dine helbredsforhold, som kommer frem i forbindelse med forsøget, er omfattet af tavshedspligt.** Det samme gælder øvrige rent private forhold og andre fortrolige oplysninger om dig.
- **Opbevaring af oplysninger om dig, herunder oplysninger i/fra dine blodprøver og væv, sker efter reglerne i lov om behandling af personoplysninger og sundhedsloven.**
- **Du har mulighed for at få aktindsigt i forsøgsprotokoller** efter offentlighedslovens bestemmelser. Det vil sige, at du kan få adgang til at se alle papirer vedrørende din deltagelse i forsøget, bortset fra de dele, som indeholder forretningshemmeligheder eller fortrolige oplysninger om andre.
- **Du har mulighed for at klage og få erstatning** efter reglerne i lov om klage- og erstatningsadgang inden for sundhedsvæsenet (se afsnittet om klagemuligheder).

For børn, voksne inhabile, bevidsthedsslørede og lignende gælder der særlige regler.

Det er frivilligt at deltage

Det er altid helt frivilligt at deltage i et sundhedsvidenskabeligt forsøg. Lægen kan derfor ikke fortælle dig, om du skal deltage eller ej – det er din helt egen beslutning.

Før du tager stilling til, om du vil deltage i et forsøg, skal du have både mundtlig og skriftlig information om forsøget. Den mundtlige information gives af den eller de personer, der har ansvar for forsøget, og det er vigtigt, at du benytter lejligheden til at stille spørgsmål, hvis du er i tvivl. Du har ret til at have en bisidder (pårørende, ven eller bekendt) med til samtalen om forsøget, så I er to til at stille spørgsmål og tale om din eventuelle deltagelse bagefter. Du har også krav på betænkningstid, før du beslutter dig – som regel mindst 24 timer.

Vælger du at sige ja til at blive forsøgsperson, skal du skrive under på, at du vil deltage, og du skal skrive under på, at du har modtaget både mundtlig og skriftlig information om forsøget. Men selv om du har skrevet under på, at du vil deltage, kan du altid træde ud af forsøget igen. Hvis du træder ud af forsøget, vil forskerne ofte gerne vide hvorfor, af hensyn til resultaternes pålidelighed, men du er ikke forpligtet til at oplyse det. Hvis du deltager som patient og træder ud af forsøget, vil du stadig modtage den behandling, som du ville have fået, hvis du ikke havde deltaget i forsøget. Du mister altså ingen rettigheder som patient ved at deltage i et forsøg.

Forskellige typer af forsøg

Et forsøg kan fx dreje sig om, at man afprøver et lægemiddel, noget medicinsk udstyr eller måske en ny behandlingsmetode, fx et forsøg, hvor man opererer på en anden måde end hidtil.

Når man afprøver et lægemiddel på mennesker, er det ofte for at undersøge lægemidlets effekt og sikkerhed og for at opdage eventuelle bivirkninger.

Forsøg med medicinsk udstyr drejer sig ofte om udstyrets sikkerhed og ydeevne. Medicinsk udstyr er udstyr, der bruges til at undersøge, behandle eller lindre sygdom (fx sprøjter, operationsudstyr, plastre, pacemakere eller hofteproteser).

Afprøvning af lægemidler og medicinsk udstyr kræver en godkendelse fra både Sundhedsstyrelsen og en videnskabsetisk komité. Forsøg, der ikke involverer lægemidler skal udelukkende godkendes af det videnskabsetiske komitésystem.

Du skal have skriftlig information om

- At du anmodes om at deltage i et sundhedsvidenskabeligt forsøg, og at du modtager ikke-almindelig behandling.
- Formålet med forsøget, og hvordan det udføres.
- (I tilfælde af lægemiddelafprøvninger:) Hvilke godkendte og ikke godkendte lægemidler, der anvendes.
- Eventuel tilfældig fordeling mellem flere forskellige behandlingsformer, hvoraf én kan være behandling med et uvirksomt præparat (placebo), hvis der ikke findes en standardbehandling.
- Risici, bivirkninger og ulemper ved at deltage i forsøget.
- Nytten af forsøget.
- Hvis der i forbindelse med forsøget udtages biologisk materiale (fx blod eller væv), der opbevares.
- (Hvis du deltager som patient:) Hvad der er almindelig behandling, om der er andre mulige behandlingsmetoder, og hvilken behandling du vil få, hvis du vælger at sige nej til at deltage i forsøget. Du skal også have oplyst, hvis der anvendes oplysninger fra din patientjournal.
- At oplysninger fra din patientjournal, rent private forhold og andre fortrolige oplysninger kan videregives til og behandles af personer, som skal foretage en lovpligtig kvalitetskontrol af forsøget.
- Under hvilke omstændigheder du kan udelukkes fra forsøget.
- Under hvilke omstændigheder forsøget kan blive afbrudt.
- Navn, adresse og telefonnummer på forsøgets kontaktperson.
- Eventuel økonomisk støtte, som forskeren modtager fra private virksomheder og fonde.
- Eventuelt vederlag for deltagelse og oplysning om beskatning heraf.

Den skriftlige information skal udleveres i god tid, så vidt muligt 24 timer inden du tager stilling til, om du vil deltage.

Krav til forskerne

Der stilles uddannelsesmæssige krav til den ansvarlige for forsøget afhængigt af forsøgets indhold. Den ansvarlige skal have ret til at udøve forskning – det kan fx være via en ansættelse som forsker, og vedkommende skal også have erfaring med at behandle patienter fra fx en hospitalsafdeling.

Hvis der er tale om forsøg med lægemidler eller medicinsk udstyr, skal den ansvarlige have en uddannelse som læge eller (hvor det er relevant) tandlæge. Hvis deltagerne under forsøget får alvorlige bivirkninger, eller der sker alvorlige hændelser, skal den forsøgsansvarlige omgående underrette den videnskabsetiske komité. Hvis der er tale om forsøg med lægemidler, skal Sundhedsstyrelsen også underrettes.

Vederlag, skattepligt mv.

I visse situationer betales der vederlag til forsøgspersoner. Der kan fx være tale om tabt arbejdsfortjeneste, transportgodtgørelse, ulempegodtgørelse eller andet. Det vil fremgå af deltagerinformationen, om der gives vederlag i forbindelse med deltagelse i forsøget, og om det er skattepligtigt.

Klage- og erstatningsmuligheder

Hvis du som forsøgsperson bliver udsat for en utilfredsstillende behandling eller kommer til skade, er der forskellige klage- og erstatningsmuligheder.

Ved godkendelse af forsøget sikrer den videnskabsetiske komité, at der er en forsikrings- og erstatningsordning for forsøgspersonerne.

Ønsker du at søge erstatning for en skade i forbindelse med et forsøg inden for sundhedsvæsenet, skal du anmelde skaden til:

Patienterstatningen
Hjemmeside: www.patienterstatningen.dk
E-mail: pebl@patienterstatningen.dk
Telefon: +45 33 12 43 43

Ordningen dækker også lægemiddelskader. Raske forsøgspersoner er også omfattet af reglerne om arbejdsskadesikring.

Er forsøget uden for det område, som Patienterstatningen dækker, skal der være tegnet en særskilt forsikring. Sådanne erhvervsansvarsforsikringer dækker normalt efter

almindelige erstatningsretlige regler. Du kan få yderligere information af den ansvarlige for forsøget. I tvivlstilfælde kan du henvende dig til sekretariatet i den videnskabsetiske komité, der dækker din region.

Du har mulighed for at klage over den sundhedsfaglige behandling i forsøget. Du kan sende klagen til:

Styrelsen for Patientsikkerhed
Hjemmeside: www.stps.dk
E-mail: stps@stps.dk
Telefon: +45 72 28 66 00

Du kan evt. få hjælp hertil fra regionernes patientvejledere.
Se nærmere på www.sundhed.dk.

Hvis du vil vide mere

Vi håber, at du har fået et tilstrækkeligt indblik i, hvad det vil sige at deltage i et forsøg, og føler dig rustet til at tage beslutningen om din eventuelle deltagelse. Hvis du vil vide mere om det at være forsøgsperson, er du meget velkommen til at kontakte sekretariatet i en af de regionale videnskabsetiske komiteer. Du kan også finde flere oplysninger og spørgsmål og svar på www.nvk.dk.

De Videnskabsetiske Komiteer for Region Hovedstaden (6 komiteer)
Tlf.: +45 38 66 63 95
E-mail: vek@regionh.dk
Hjemmeside: www.regionh.dk/vek

De Videnskabsetiske Komiteer for Region Syddanmark (2 komiteer)
Tlf.: + 45 76 63 82 21
E-mail: komite@rsyd.dk
Hjemmeside: www.regionsyddanmark.dk/komite

Den Videnskabsetiske Komité for Region Nordjylland
Tlf.: +45 97 64 84 40
E-mail: vek@rn.dk
Hjemmeside: www.vek.rn.dk

Den Videnskabsetiske Komité for Region Sjælland
Tlf.: +45 93 56 60 00
E-mail: RVK-sjaelland@regionsjaelland.dk
Hjemmeside: www.regionsjaelland.dk/sundhed/forskning/forfagfolk/videnskabsetisk-komite/Sider/default.aspx

De Videnskabsetiske Komiteer for Region Midtjylland (2 komiteer)
Tlf.: +45 78 41 01 83
/ +45 78 41 01 82 / +45 78 41 01 81
E-mail: komite@rm.dk
Hjemmeside: www.komite.rm.dk

Den Nationale Videnskabsetiske Komité
Tlf.: +45 72 26 93 70
E-mail: kontakt@nvk.dk
Hjemmeside: www.nvk.dk

Forsøgspersoners rettigheder

i et sundhedsvidenskabeligt forskningsprojekt

Som deltager i et sundhedsvidenskabeligt forskningsprojekt skal du vide, at:

- din deltagelse i forskningsprojektet er helt frivillig og kun kan ske efter, at du har fået både skriftlig og mundtlig information om forskningsprojektet og underskrevet samtykkeerklæringen.
- du til enhver tid mundtligt, skriftligt eller ved anden klar tilkendegivelse kan trække dit samtykke til deltagelse tilbage og udtræde af forskningsprojektet. Såfremt du trækker dit samtykke tilbage påvirker dette ikke din ret til nuværende eller fremtidig behandling eller andre rettigheder, som du måtte have.
- du har ret til at tage et familiemedlem, en ven eller en bekendt med til informationssamtalen.
- du har ret til betænkningstid, før du underskriver samtykkeerklæringen.
- oplysninger om dine helbredsforhold, øvrige rent private forhold og andre fortrolige oplysninger om dig, som fremkommer i forbindelse med forskningsprojektet, er omfattet af tavshedspligt.
- opbevaring af oplysninger om dig, herunder oplysninger i dine blodprøver og væv, sker efter reglerne i lov om behandling af personoplysninger og sundhedsloven.
- der er mulighed for at få aktindsigt i forsøgsprotokoller efter offentlighedslovens bestemmelser. Det vil sige, at du kan få adgang til at se alle papirer vedrørende din deltagelse i forsøget, bortset fra de dele, som indeholder forretningshemmeligheder eller fortrolige oplysninger om andre.
- der er mulighed for at klage og få erstatning efter reglerne i lov om klage- og erstatningsadgang inden for sundhedsvæsenet. Hvis der under forsøget skulle opstå en skade kan du henvende dig til Patienterstatningen, se nærmere på www.patienterstatningen.dk.

De Videnskabsetiske Komiteer for Region Hovedstaden (6 komiteer)
Tlf.: +45 38 66 63 95
E-mail: vek@regionh.dk
Hjemmeside: www.regionh.dk/vek

De Videnskabsetiske Komiteer for Region Syddanmark (2 komiteer)
Tlf.: + 45 76 63 82 21
E-mail: komite@rsyd.dk
Hjemmeside: www.regionsyddanmark.dk/komite

Den Videnskabsetiske Komité for Region Nordjylland
Tlf.: +45 97 64 84 40
E-mail: vek@rn.dk
Hjemmeside: www.vek.rn.dk

Den Videnskabsetiske Komité for Region Sjælland
Tlf.: +45 93 56 60 00
E-mail: RVK-sjaelland@regionsjaelland.dk
Hjemmeside: www.regionsjaelland.dk/sundhed/forskning/forfagfolk/videnskabsetisk-komite/Sider/default.aspx

De Videnskabsetiske Komiteer for Region Midtjylland (2 komiteer)
Tlf.: +45 78 41 01 83
/ +45 78 41 01 82 / +45 78 41 01 81
E-mail: komite@rm.dk
Hjemmeside: www.komite.rm.dk

Den Nationale Videnskabsetiske Komité
Tlf.: +45 72 26 93 70
E-mail: kontakt@nvk.dk
Hjemmeside: www.nvk.dk

Dette tillæg er udarbejdet af det videnskabsetiske komitésystem og kan vedhæftes den skriftlige information om det sundhedsvidenskabelige forskningsprojekt. Spørgsmål til et konkret projekt skal rettes til projektets forsøgsansvarlige. Generelle spørgsmål til forsøgspersoners rettigheder kan rettes til den komité, som har godkendt projektet.
